# Turbulent Flow in Street Canyons: A Complexity Approach

**DOI:** 10.3390/e27050488

**Published:** 2025-04-30

**Authors:** Csanád Árpád Hubay, Bálint Papp, Tamás Kalmár-Nagy

**Affiliations:** Department of Fluid Mechanics, Faculty of Mechanical Engineering, Budapest University of Technology and Economics, 1111 Budapest, Hungary; papp.balint@gpk.bme.hu (B.P.); kalmar.nagy.tamas@gpk.bme.hu (T.K.-N.)

**Keywords:** street canyon, turbulence, quadrant method, word statistics, entropy, Markov statistics

## Abstract

Velocity measurements and simulations in an idealized urban environment were studied, focusing on turbulent flow over street canyons. Time series of fluctuating velocities were considered as marked point processes, and the distribution of mean residence times was characterized using a lognormal fit. The quadrant method was applied to transform time series into symbolic sequences, enabling the investigation of their information content. By analyzing word frequency and normalized entropy levels, we compared measured and simulated sequences with periodic symbol sequences with and without noise. Our results indicate that noisy periodic sequences exhibit entropy distributions qualitatively similar to those of the measured and simulated data. Surrogate sequences generated using first-, and higher-order Markov statistics also displayed similarity. Higher-order Markov chains provide a more accurate representation of the information content of velocity fluctuation series. These findings contribute to the comparison of experimental and simulation techniques in the investigation of turbulence.

## 1. Introduction

Air pollution is closely linked to public health and climate issues. Understanding pollutant dispersion in urban environments is key for development of turbulence models capturing this phenomenon. Atmospheric boundary layer turbulence is a complex process of interacting coherent structures (spatial regions where flow properties are strongly correlated); thus, we seek to identify tools to detect and characterize coherent structures.

Robinson [1] presented a review of major findings and challenges concerning coherent structures in turbulent boundary layers, specifically those developing over flat surfaces with zero pressure gradient and at relatively low Reynolds numbers (Re < 5000). A study by Kline et al. [2] revealed the presence of near-wall low-speed “streaks” originating in the laminar sublayer. The dynamic sequence involving the generation, upward movement, and disintegration of these structures is widely referred to as the “bursting” process. Offen and Kline [3] characterized this behavior as a “shear layer interface sandwiched between an upstream, high-speed sweep and a downstream, low-speed ejection”.

Bursts are essential contributors to momentum transfer over short durations due to their dominant role in producing turbulent kinetic energy within shear-driven flows. The study of burst-related phenomena generally focuses on three main areas: (i) techniques for detecting burst intervals, (ii) the formulation of appropriate scaling laws for burst duration, and (iii) exploring how bursts relate to coherent structures. An overview of research addressing these themes was provided by Metzger et al. [4]. In [5], Mahrt investigated intermittent turbulence bursts in the atmospheric boundary layer, emphasizing that higher-order statistics and event-based statistical analysis is needed to characterize non-Gaussian behavior of burst-dominated flows. Later, Mahrt focused on turbulence under stable and stratified conditions [6] and noted that turbulence is patchy and intermittent, especially in strongly stratified conditions.

Renewal processes are important to our understanding of intermittent and burst-like transport phenomena. Higbie [7] and Danckwerts [8] showed that interfacial transfer cannot be understood purely from a static perspective; rather, stochastic and dynamic surface processes should be used (eddies rise to or fall from the interface, carrying new fluid volumes). Perlmutter [9] introduced realistic eddy age/lifetime distributions into surface renewal models.

Katul et al. [10] applied renewal theory to model passive scalar transport in turbulent flows. Paradisi and collaborators [11,12,13] further incorporated renewal concepts to describe scalar dissipation, turbulent structure, and memory effects in stratified boundary layers. Cesari et al. [14] and Luo et al. [15] extended renewal-based models to environmental turbulence and wind energy applications, respectively, highlighting the model’s versatility across complex boundary-layer phenomena.

Using direct numerical simulations, Itano and Toh [16] examined burst dynamics in turbulent channel flows and identified a traveling wave solution corresponding to a saddle point in the low-dimensional near-wall system, shedding light on the self-sustaining nature of wall turbulence. Similarly, Jiménez et al. [17] employed direct numerical simulations to investigate near-wall turbulence in Couette and Poiseuille flows, revealing that intermittent bursting is primarily associated with vortex-dominated quiescent structures.

Kawahara and Kida [18] discovered an exact time-periodic solution for Couette flow, offering a simplified model for the bursting process. The nonlinear growth and transition mechanisms in Couette flow were further explored by Cherubini and De Palma [19], focusing on the evolution of optimal disturbances. Bomminayuni and Stoesser [20] analyzed dominant turbulent events, such as sweeps and ejections, and their contributions to Reynolds stress, also quantifying turbulence anisotropy in rough-bed open-channel flows.

Quantitative insights into fluctuating velocity components were historically obtained through the development of conditional sampling methods, designed to enhance flow visualization by providing statistical context. Foundational contributions to this approach include the early work of Wallace [21] and Willmarth [22].

The *quadrant method* provides a useful framework for analyzing turbulent flows by transforming velocity time series into symbolic sequences, enabling the investigation of their information content. The streamwise and vertical velocity components are decomposed into their mean and fluctuating parts. The signs of the fluctuating streamwise and vertical components distinguish four distinct quadrants, each representing different turbulent events—*outward interaction*, corresponding to the upward motion of faster-than-average fluid; *ejection*, representing the upward motion of slower-than-average fluid; *inward interaction*, indicating the downward motion of slower-than-average fluid; and *sweep*, describing the downward motion of faster-than-average fluid.

From the standpoint of turbulent momentum exchange, ejection and sweep events are essential mechanisms that facilitate the mixing of slow-moving near-wall fluid with faster fluid from above. An extensive account of quadrant analysis and its applications is presented by Wallace [23]. Further discussion on conditional sampling approaches, including variable-interval time averaging, can be found in Antonia et al. [24].

Quadrant-based methods have been employed for various turbulent flows, including river hydraulics, the atmospheric boundary layer, and wind tunnel studies. For instance, Kirkbride et al. [25] performed simultaneous measurements of vertical and streamwise velocities at multiple points above a gravel riverbed. By applying a simplified quadrant analysis, they categorized turbulence states and analyzed their transitions using a Markov chain model. Their results revealed the presence of large-scale structures.

Using a similar experimental setup, Buffin-Bélanger et al. [26] corroborated the role of large-scale coherent structures in shaping river turbulence. They demonstrated that quadrant events frequently occur in cyclic or oscillatory groupings, likely driven by the passage of alternating high- and low-momentum fluid regions.

To further understand the spatiotemporal properties of such coherent structures, Roy et al. [27] utilized an array of electromagnetic flow meters to characterize their scale, lifespan, and dynamics. Their statistical analysis of burst durations revealed that lab-scale observations align closely with the scaling behavior of natural river flows.

Field measurements have also identified coherent structures in atmospheric flows. Finnigan [28] demonstrated that large-scale coherent structures dominate flow dynamics above plant canopies. Quadrant analysis showed that the sweep quadrant contributes most to momentum flux, as fast downward gusts penetrate lower boundary layer levels. In urban settings, Christen et al. [29] used anemometers in a vertical grid across an urban canyon to study momentum and scalar transport. They found that sweep events dominate transport in the upper canyon, while ejection events follow sweeps in a cyclical pattern. Nelson et al. [30] demonstrated that all four quadrants contribute to momentum transfer near the roof level within street canyons. They introduced the concept of a disorganized canopy layer, where the aerodynamic influence of individual buildings outweighs that of broader atmospheric flow structures.

Since direct turbulence measurements in urban environments are costly and complex, wind tunnel models are widely used [31]. Kukačka et al. [32] investigated flow and tracer gas concentration above an X-shaped street intersection, analyzing the dominant role of sweep events in impulse transfer. Nosek et al. [33] applied quadrant analysis to study turbulence above a 3D street canyon, showing that:

- Sweeps correlate with entrainment of clean air;
- Ejections are associated with ventilation of polluted air;
- Coherent structures passing over the canyon drive this process.

Kellnerová et al. [34] examined the velocity field within a two-dimensional idealized street canyon using Particle Image Velocimetry. Their findings indicate that the street canyon vortex is frequently disturbed, a process which can significantly influence the efficiency of urban ventilation. Additionally, Di Bernardino et al. [35] reported that the shear layer above the canyon roof exhibits a periodic flapping motion.

Conditional sampling techniques are commonly used for event detection in turbulent flows. This inspired our research into symbolic turbulence data analysis. A comprehensive review of symbolic analysis and its applications is provided by Daw et al. [36]. The key step in symbolic analysis involves converting the original dataset into a sequence of symbols. For continuous datasets, this is typically achieved by partitioning the range of observed values into a finite number of discrete regions. Each data point is then replaced by the symbol corresponding to its respective region. This process, known as coarse graining or quantization, reduces high-resolution data to a lower-resolution representation.

Lehrman and Rechester [37] developed a symbolic cycle extraction method for turbulent fluctuations in pipe flows. If simultaneous velocity component measurements are available, quadrant-based symbolization is a viable approach.

Daw et al. [38] studied pressure fluctuations from air bubbles released through a nozzle into a water tank, aiming to detect periodic behavior in a two-phase fluid system under varying Reynolds numbers. They converted the time series into binary symbol sequences using the dataset median as a threshold and introduced a modified Shannon entropy, referred to as *normalized entropy*, to evaluate the informational complexity of the symbolized data. To characterize the bubble formation process, they used word statistic histograms, where “words” represented smaller partitions of the symbolic sequence.

Using a comparable symbolic framework, Finney et al. [39] analyzed heat release patterns across combustion engine cycles. Their results indicated that normalized entropy reaches a minimum at an optimal word length, beyond which the symbolic sequences either fail to encapsulate meaningful temporal structure (if too short) or become dominated by noise and limited sample size (if too long). This highlights the delicate balance between resolution and statistical reliability in symbolic analysis.

Keshavarzi et al. [40] investigated the turbulent bursting process and its link to sediment transport using velocity measurements from an open water channel. By applying the quadrant method, they encoded instantaneous vertical and streamwise velocity components into discrete symbolic states. The sequence of transitions between these states was modeled using a first-order Markov chain.

Gheisi et al. [41] expanded upon this approach by exploring the three-dimensional bursting process near the bed of a settling chamber. Using synchronized measurements of all three velocity components, they developed the *octant method*, which symbolized the data according to the sign of velocity fluctuations along all three axes. Their analysis confirmed that a first-order Markov model was sufficient to capture the dominant dynamics of the bursting process. A similar Markov-based approach was employed by Jin et al. [42], who simulated particle deposition near the wall by incorporating flow eddies, effectively modeling the impact of coherent structures on heavy particle trajectories.

In a wind tunnel study aimed at understanding the temporal organization of turbulence in urban-like environments, Kalmár-Nagy and Varga [43] analyzed two-component velocity signals. Their methodology, closely followed in the present work, treated velocity fluctuations as a marked point process and used the quadrant method to construct symbolic sequences. Entropy analysis revealed that these sequences exhibited traits similar to noisy periodic signals, necessitating higher-order Markov chains to accurately capture the information content. Furthermore, performing quadrant analysis with rotated coordinate systems minimized entropy in the principal axes system of the velocity fluctuation cloud, suggesting a fundamental structure in turbulence dynamics. In this paper, we extend the analysis of the streamwise and vertical velocity components by including both measurements and simulations of street canyon flows, i.e., an idealized urban environment. The novelty of our work lies in the direct comparison between experimental and simulated symbolic sequences.

Further contributions by Chowdhuri et al. [44,45,46] provided detailed persistence-based analyses of velocity and temperature fluctuations in convective turbulence. Their work explored intermittent heat transport, Reynolds stress anisotropy, and temperature variability during gust front events, offering insights into the structure of atmospheric turbulence.

The paper is organized as follows. Section 2 details the experimental and numerical setups, along with the signal acquisition and processing methodologies. In Section 3, we explore statistical characteristics of velocity fluctuations, comparing the experimental observations with simulation results. Section 4 introduces a quadrant-based symbolic encoding of both datasets and the marked point process used in the study. Section 5 delves into word statistics derived from the symbolic sequences and quantifies their information content through normalized entropy. In Section 6, we examine the Markovian properties of both the symbolic series and the quadrant-based marked point process. We also compare these datasets with surrogate sequences that mimic noisy periodic behavior and demonstrate how higher-order Markov models can replicate the symbolic data’s informational structure. Section 7 focuses on the two-dimensional distribution of velocity fluctuations, and Section 8 summarizes the key findings.

## 2. Data

Street canyons are widely used as a minimalistic model of the urban environment. The literature of street canyon flows is reviewed extensively by Vardoulakis et al. [47], Ahmad et al. [48], Li et al. [49], Yazid et al. [50], Zhang et al. [51], and Voordeckers et al. [52]. In the present study, the flow field within and above a series of parallel street canyons with uniform building height and perpendicular wind conditions were analyzed via wind tunnel experiments and Computational Fluid Dynamics (CFD) simulations.

### 2.1. Wind Tunnel Measurements

#### 2.1.1. Experimental Setup

**Figure 1:**
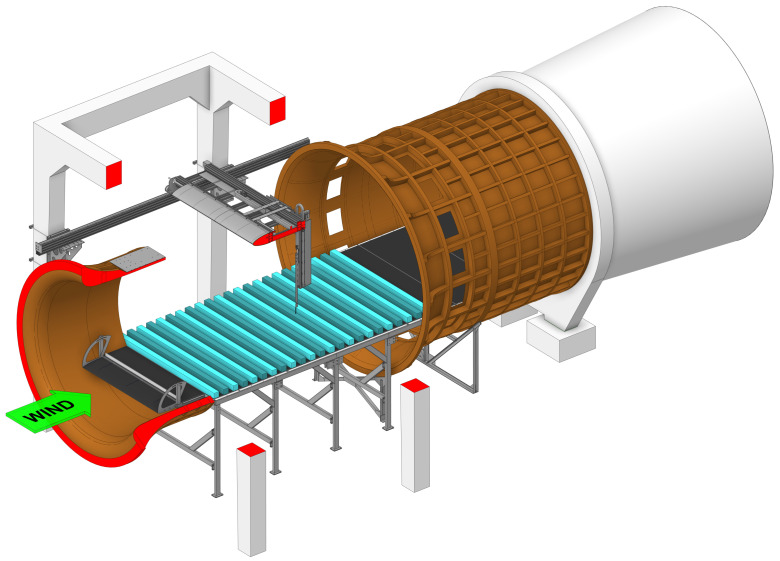
The wind tunnel setup used for the acquisition of the experimental time series.

The wind tunnel experiments were performed using the closed-circuit horizontal (Göttingen-type) wind tunnel of the Theodore von Kármán Wind Tunnel Laboratory at the Department of Fluid Mechanics of the Budapest University of Technology and Economics. The wind tunnel has a circular cross-section of 2.6 m in diameter at the open test section of 3.8 m in length, and it is equipped with a 2.5 m wide horizontal table (Figure 1).

**Figure 2:**
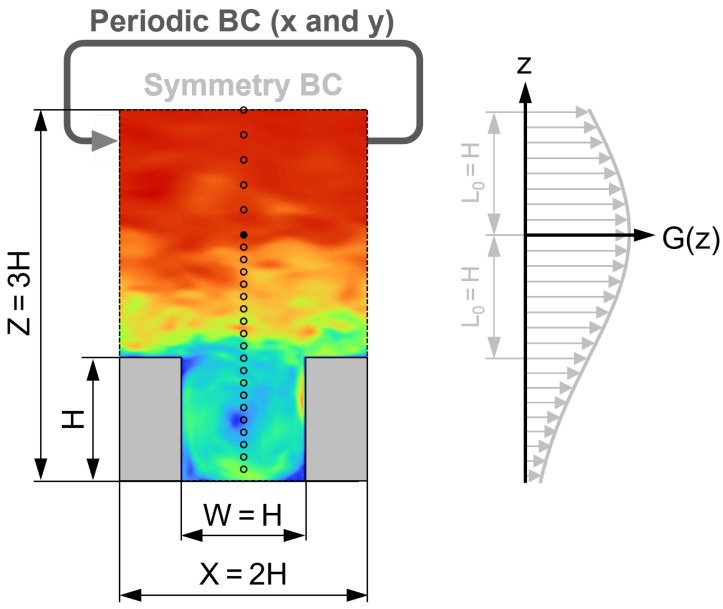
Layout and dimensions of the computational domain, containing a street canyon with H=0.1 m building height. The size of the computational domain in the *y* direction is Y=1.04H. G(z) denotes the vertical distribution of the driving force of the TWF model with $L_{0}/H=1.0$ radius. The reference location, i.e., the center of the TWF propulsion at $z_{0}/H=2.0$, is denoted by a filled marker. The sampling points used for recording the velocity time series are denoted by hollow markers. The background is colored by the instantaneous velocity magnitude.

In total, 23 rows of prismatic building blocks were constructed from styrofoam and were mounted to the floor of the test section, forming 22 consecutive street canyons, with their axis oriented perpendicular to the incoming flow. The height-to-width aspect ratio of the canyons was H/W=1.0, with the roof height H=0.1 m being equal to both the street width *W* and the building breadth *B*. The dimensions of the geometry are shown in detail in Figure 2. The incoming flow of the wind tunnel was homogeneous with a free-stream velocity of $u_{\infty }=9$ m/s, and below 1% turbulence intensity. No additional roughness elements were placed upstream of the building models; this way, the boundary layer could adapt to the investigated building configuration. It was shown by Papp et al. [53,54] that the flow field is fully developed after the first 11 canyons.

The Reynolds number based on the building height and the free-stream velocity yields $Re=u_{\infty }H/\nu =5\cdot 10^{4}$, in which ν is the kinematic viscosity of air. This value is high enough for the velocity field to be considered independent of the Reynolds number according to the results of Chew et al. [55] for H/W=1.0 street canyons. Papp et al. [53] have pointed out that the velocity field showed indeed very little variation as the function of the Reynolds number in the $Re=2.5\cdots 7.5\cdot 10^{4}$ range ($u_{\infty }=4.5\ldots 13.5$ m/s); therefore, the results obtained in model scale can be considered relevant in full scale as well.

#### 2.1.2. Measurement Techniques and Signal Processing

The time histories of the streamwise and vertical velocity components denoted by u(t) and w(t) hereinafter, were measured simultaneously, using a two-component Laser Doppler Anemometer by TSI. For details of the measurement instrumentation, the reader is referred to Papp et al. [53,54].

The velocity sampling points were located in a vertical array in the middle of the 11th street canyon. The height of the *i*th measurement point can be calculated as $z_{i}=iH/10$, with i=1,2,…,19,20,22,24,26,28,30.

The velocity measurement at each point took 150 s with a sampling frequency of 100…1000+ Hz, depending on how effectively the seeding particles released upstream could reach each point. The free-stream velocity ($u_{\infty }=9$ m/s) of the wind tunnel was monitored using a Pitot-static probe, and its minor changes were compensated for during the normalization process. The absolute measurement uncertainty of the LDA system was 0.1 m/s at 5 m/s flow velocity.

Generally, the data sampling frequency in LDA measurements is not constant. Moreover, the so-called coincidence mode of the LDA system was not enabled in order to achieve as high sampling rate as possible; therefore, the LDA bursts corresponding to the u(t) and w(t) velocity components were not recorded at the same time instances. To overcome the temporal irregularity of the measured data series, the time series between 4 s and 34 s were re-sampled to the temporal resolution of the CFD time series using linear interpolation for further data processing (see Table 1 and Section 2.2.4).

### 2.2. Computational Fluid Dynamics Simulations

#### 2.2.1. Boundary Conditions and Numerical Mesh

The computational domain used in the numerical simulations consists of a three-dimensional section of a single street canyon (see Figure 2).

The size of the computational domain is X×Y×Z = 2H×2.0833H×3H. On the lateral boundaries, periodic boundary conditions (BCs) are assumed (in both *x* and *y* directions), and at the top of the domain, slip symmetry (zero gradient BC) is assumed. At the solid surfaces, i.e., the ground and the building walls, smooth, no-slip walls are applied.

The computational domain was discretized using equidistant Cartesian meshes of three different spatial resolutions H/Δx= 22, 32, and 48. Note that according to Xi and Castro [56], a mesh uniformly resolving the characteristic length by 16 cells can be considered sufficiently dense for capturing the large-scale eddies governing the dispersion processes in urban-like obstacle arrays using large eddy simulation (LES)—the same turbulence model as used for the present investigation. The mesh dependence of the results was assessed based on the descriptive statistics (mean, standard deviation, skewness, kurtosis) as well as the residence times (Equation (6)). It was found that although the results are not independent of the mesh resolution, they show monotonous convergence. This is expected behavior, as LES run on a finer mesh can resolve more turbulent kinetic energy. In this paper, the results obtained on the finest mesh are shown only.

#### 2.2.2. Transient Wind Forcing: Modeling Large-Scale Turbulence in a Small Periodic Domain

Importantly, the main driving force of atmospheric flows is the momentum exchange at the top of the atmospheric boundary layer (ABL), resulting in the formation of large-scale eddies, which cause sudden changes in the wind direction and magnitude near the surface. The review article by [51] highlighted that accurately modeling the resultant time-varying inflow conditions can have a significant effect on the governing flow regimes observed in street canyons; therefore, it is important to consider the effects of large-scale turbulence in microscale meteorological models.

The Transient Wind Forcing (TWF) method was introduced by Kristóf et al. [57] to model the effects of the changing wind direction and magnitude in a small periodic computational domain using velocity time series recorded in field experiments or mesoscale simulations. Moreover, their model was capable of tracking particle trajectories in a horizontally limitless space based on the periodic velocity field. Papp et al. [53] added the capability of calculating a spatially continuous concentration field derived from discrete particle tracks to the TWF model, and the latest version published by Koren and Kristóf [58] allows for even modeling thermal stratification and the Coriolis force.

According to the TWF approach, turbulence can be divided into the following categories based on the size of the flow structures:

1. Macroscopic turbulence, i.e., eddies exceeding the size of the computational domain;
2. Mesoscopic turbulence, i.e., eddies that are resolved in the LES calculations;
3. Microscopic turbulence, i.e., vortices that are smaller than the mesh resolution; therefore, their effects are modeled by the subgrid-scale stress (SGS) model.

In the TWF model, the effect of macroscopic turbulence is taken into account as a time-dependent driving force, implemented as sources terms in the *x* and *y* components of the momentum equation. The source terms are formulated as

(1) $$\begin{array}{cc} \; & S_{u}\left(z,t\right)=\rho \cdot a_{u}\left(t\right)\cdot G\left(z\right)=\rho \cdot \frac{u_{0}\left(t\right)-u\left(t\right)}{\tau (t)}\cdot e^{-\frac{1}{2}{\left(\frac{z-z_{0}}{L_{0}}\right)}^{2}}, \end{array}$$

(2) $$\begin{array}{cc} \; & S_{v}\left(z,t\right)=\rho \cdot a_{v}\left(t\right)\cdot G\left(z\right)=\rho \cdot \frac{v_{0}\left(t\right)-v\left(t\right)}{\tau (t)}\cdot e^{-\frac{1}{2}{\left(\frac{z-z_{0}}{L_{0}}\right)}^{2}}. \end{array}$$

In the above equations $S_{u}\left(z,t\right)$ and $S_{v}\left(z,t\right)$ denote the volume source intensities, ρ is the constant air density, $u_{0}\left(t\right)$ and $v_{0}\left(t\right)$ are the measured velocity time series (reference time series), u(t) and v(t) are the velocity components in the CFD model, and τ(t) is the relaxation time of the velocity control. Furthermore, *t* is time, *z* is the vertical coordinate, and $z_{0}/H=2.0$ is the reference height. As can be seen in the above formulas, the vertical distribution of the driving force is Gaussian, with its center being at $z_{0}$, and with a characteristic radius of $L_{0}/H=1.0$, shown in Figure 2. The direction of the resultant body force can take any horizontal direction, determined by the values of the so-called control terms $a_{u}\left(t\right)$ and $a_{v}\left(t\right)$, which are responsible for imposing the measured velocity time series at the reference point.

The relaxation time in the denominator of the control terms is increased exponentially within the startup transient expressed by the formula

(3) $$\tau \left(t\right)=\Delta t_{0}+\left(\Delta t-\Delta t_{0}\right)\cdot e^{-\frac{t-t_{\mathrm{start}}}{\Delta t_{0}}},$$

where $\Delta t_{0}=0.0195$ s is the propulsion time scale of the TWF model (half of the flow-through time calculated as $X/u_{0}$, in which $u_{0}=5.117$ m/s is the mean streamwise velocity at $z_{0}$), $t_{\mathrm{start}\;}$ is the starting time, and Δt is the time step size of the simulation. The latter was fixed, with its value chosen to satisfy the Courant–Friedrichs–Lewy (CFL) criterion, i.e., the maximum of the Courant number (C=uΔx/Δt) in the entire computational domain should be around 1.

An appropriately chosen relaxation time allows for the applied large eddy simulation (LES) turbulence model, to generate realistic mesoscopic turbulence around the reference point, with the large-scale trends following the velocities prescribed via $u_{0}\left(t\right)$ and $v_{0}\left(t\right)$ accurately, but it prevents the TWF driving force from acting as a direct velocity constraint, resulting in an unrealistic flow field. Imposing the reference time series recorded in the wind tunnel experiments is effectively an immersed boundary condition. Therefore, reproducing the velocity field above the reference height is out of the scope of the TWF approach; however, to allow free eddy motion, the simulation domain was kept taller.

The effects of microscopic turbulence was taken into account using the Smagorinsky–Lilly subgrid-scale stress (SGS) model [59,60], using $C_{s}=0.1$, following the recommendations of Shah [61] for a flow past a blunt obstacle.

#### 2.2.3. Solver Setup

For the numerical solution of the flow equations, Ansys Fluent [62], a general-purpose CFD solver, was utilized, applying the Bounded Central Differencing Scheme flux formulation and the SIMPLE (Semi-Implicit Method for Pressure Linked Equations) algorithm, along with the bounded second-order implicit scheme for temporal discretization. Iterative solution was carried out at each time step until the residuals of the flow equations were reduced by at least three orders of magnitude compared to the initial state, but in a maximum of 20 iterations per time step.

#### 2.2.4. Time Series Acquisition

**Figure 3:**
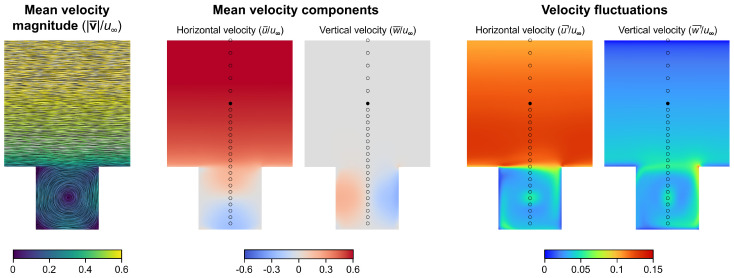
CFD results: time averaged streamlines plotted over the mean normalized velocity magnitude; mean velocity components (streamwise u(t) and vertical w(t)); and the time-averages of the velocity fluctuations. The velocity statistics are averaged over time and the spanwise direction, i.e., the length of the street canyon, and are normalized by the free-stream velocity $u_{\infty }$. (The sampling points used for recording the velocity time series are denoted by hollow markers. The filled marker denotes the center of the TWF propulsion at $z_{0}/H=2.0$.)

The time series used for the analysis of the flow field were recorded in each time step for all three velocity components, at 25 gauging points placed in a vertical array identical to that of the wind tunnel experiments described in Section 2.1.2 and shown in Figure 3. The length of the sampling interval was 34 s in each case, but the initial 4 s of the simulations were considered part of the startup transient based on the visual assessment of the time series; therefore, the t<4 s intervals were discarded. Further information about the time series is included in Table 1.

#### 2.2.5. General Description of the Flow Field

Figure 3 shows the velocity statistics. The streamlines plotted over the normalized mean velocity magnitude reveal that below roof height, a closed vortex structure (with an axis of rotation parallel to the *y* axis, rotating in the clockwise direction) is formed, called the canyon vortex. Above roof height, the velocity magnitude gradually increases in the boundary layer as we move away vertically from the buildings. As shown by the spatial distribution of the time-averaged velocity components, the gradual increment of the velocity magnitude comes from the increasing streamwise velocity (∂u/∂z>0). It is not pictured in this figure, but due to the prismatic geometry and the perpendicular wind direction, the time-average of the spanwise velocity component v(t) is zero in the entire computational domain. Importantly, the maximum normalized streamwise velocity is around 0.6, as the computational domain does not include the full depth of the atmospheric boundary layer, but uses an immersed boundary condition in the form of the TWF propulsion at $z_{0}/H=2.0$. It can also be observed in Figure 3 that the mean of the vertical velocity components w(t) are uniformly close to zero above roof height. Within the canyon, the formation of the clockwise-rotating canyon vortex can also be identified in the contour plots of the mean $\bar{u}$ and $\bar{w}$ velocity components.

It can also be observed in Figure 3 that above roof height, the streamwise velocity fluctuations are 3–4 times higher than the vertical ones, which is more than the 2:1 ratio expected in atmospheric boundary layer flows, and might be attributed to the fact that in the TWF method, the volume source terms act in the horizontal directions, but not in the vertical one; hence, the vertical velocity fluctuations recorded in the experiments are not directly imposed in the CFD model. The vertical fluctuations are further attenuated as we approach the top of the domain, where the applied symmetry boundary condition damps any fluctuations perpendicular to it. Below roof height, the magnitude of the streamwise and vertical velocity fluctuations—similarly to the time-averaged values—are close to one another. Distinct local maxima can be found at the vortex core near z/H=0.5, and at the shear layer at z/H=1.0, which is responsible for driving the cavity flow within the canyon.

## 3. Descriptive Statistics of the Experimental and Simulation Data

The basic statistics of the measured and simulated velocity components u(t) and w(t) at different locations are given in Table 2.

The mean value of the vertical velocity component w(t) is close to $\bar{w}=0$ for all heights, meaning that the data represents fluctuations rather than persistent up/down motions. Table 2 shows that the probability density histograms of u(t) and w(t) for both cases have mainly normal distribution (note that Table 2 contains the excess kurtosis values). The skewness and kurtosis values are similar to those given in Czernuszenko et al. [63]. Comparing the experiment and simulation the most significant difference can be seen at z/H=1.0, where the distribution of the simulated u(t) is more skewed towards smaller values and “skinnier” than for the experimental data. The mean of the simulated u(t) at z/H=3.0 is lower than that of the experimental data. Furthermore, the range of the measured w(t) is bigger and the corresponding distribution is less skewed than for the simulated vertical velocity component.

The fluctuating components of the streamwise and vertical velocity, $u^{\prime }$ and $w^{\prime }$, respectively, are

(4) $$u^{\prime }=u\left(t\right)-\bar{u},\;w^{\prime }=w\left(t\right)-\bar{w}.$$

where $\bar{u}$ and $\bar{w}$, i.e., the time-averaged velocity components over an observation period $T_{p}$ are given by

(5) $$\bar{u}=\frac{1}{T_{p}}\int_{t}^{t+T_{p}}u\left(\tau \right)d\tau ,\;\bar{w}=\frac{1}{T_{p}}\int_{t}^{t+T_{p}}w\left(\tau \right)d\tau .$$

The velocity fluctuations $u^{\prime }$ and $w^{\prime }$ can be analyzed by identifying the location of their sign changes (note that the $u^{\prime }<0↔u^{\prime }>0$ and $w^{\prime }<0↔w^{\prime }>0$ switches are treated separately). These sign changes are called *events*. The set of times $\left\{t_{i}\right\}$ at which these events occur provides a so-called random point process [64,65,66]. Other examples of random point processes are neuronal spike patterns (spike trains) and customer arrival times.

**Figure 4:**
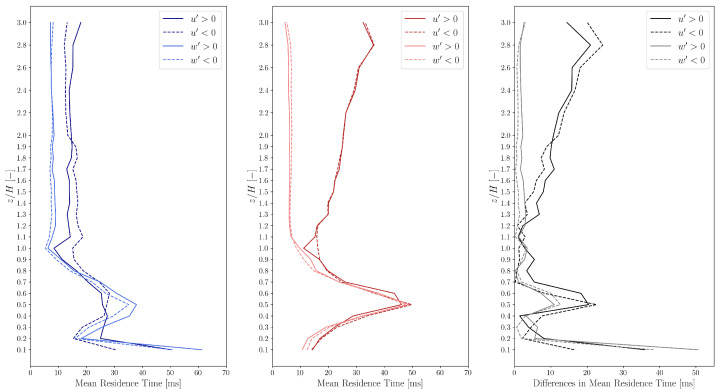
Residence time statistics of states $u^{\prime }>0$, $u^{\prime }<0$, $w^{\prime }>0$, $w^{\prime }<0$ in the experimental (**left**, blue) and simulation (**middle**, red) data. The differences (**right**, black) between the experimental and simulation data are also shown.

Let us define the residence times

(6) $$T_{i}=t_{i}-t_{i-1},\;i>1,$$

to express the duration of an event, i.e., the duration the system spends in state $u^{\prime }>0$, $u^{\prime }<0$, $w^{\prime }>0$ or $w^{\prime }<0$. The mean residence times $T_{mean}$ as a function of height are shown in Figure 4.

The mean residence times are between 5 and 60 ms for both the experimental and simulation data. However, the simulation data show high discrepancies in the residence times of the horizontal fluctuations $u^{\prime }$ compared to the experimental results.

In general, the values of $T_{mean}$ for $w^{\prime }<0$ and $w^{\prime }>0$ are smaller than those for $u^{\prime }<0$ and $u^{\prime }>0$ for locations above the height of the building, showing that at these locations the fluctuation of the vertical velocity component w(t) is more rapid than that of the horizontal velocity component u(t). However, close to the ground (only for the experimental data) and in the vortex core the values of $T_{mean}$ for the measured $w^{\prime }<0$ and $w^{\prime }>0$ are larger than those for $u^{\prime }<0$ and $u^{\prime }>0$.

The mean residence times of the vertical fluctuations $w^{\prime }$ show acceptable correspondence above roof height. Furthermore, the simulations yield mean residence times corresponding to the streamwise velocity substantially larger than the experimental observations, which can be attributed to the fact that the TWF control filters the streamwise velocity fluctuations above the frequency corresponding to the propulsion time scale ($\Delta t_{0}=0.0195$ s).

## 4. Statistics of “Quadrantified” Experimental and Simulation Data

The *quadrant method* distinguish four different states based on the signs of $u^{\prime }$ and $w^{\prime }$, such as

- Outward interaction (Q1): $u^{\prime }>0,\;w^{\prime }\geq 0$;
- Ejection (Q2): $u^{\prime }\leq 0,\;w^{\prime }>0$;
- Inward interaction (Q3): $u^{\prime }<0,\;w^{\prime }\leq 0$;
- Sweep (Q4): $u^{\prime }\geq 0,\;w^{\prime }<0$.

**Figure 5:**
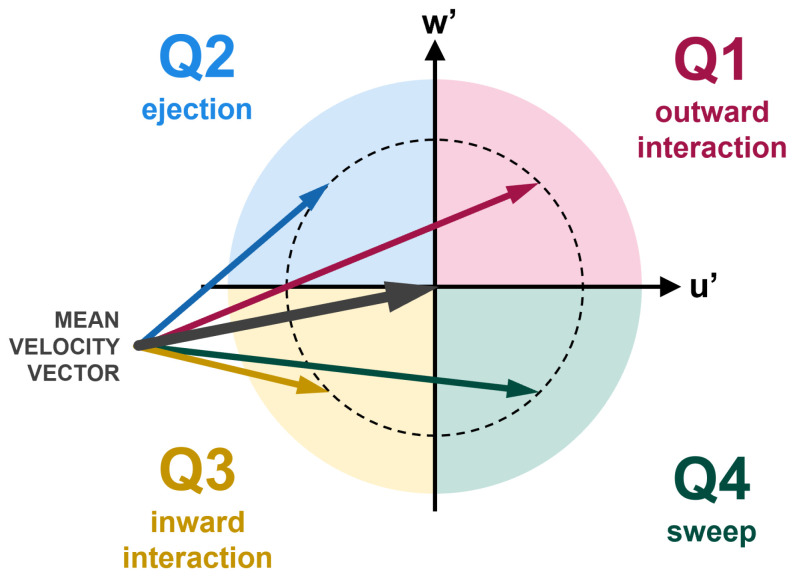
Visualization of the four quadrants.

Note that as the mean of the vertical velocity components are close to zero ($\bar{w}=0$) in all 25 gauging points (see Figure 3), it can be stated that Q1 and Q2 are associated with upward motion and Q3 and Q4 are associated with downward motion at those specific locations. The visualization of the four quadrants is shown in Figure 5.

We now apply the quadrant method to the experimental and CFD data. To the joint velocity fluctuation pair $v=(u^{\prime },w^{\prime })$, we assign a quadrant index according to

(7) $$Q\left(v\right)=\left\{\begin{array}{cc} 1, & u^{\prime }>0,\;w^{\prime }\geq 0\\ 2, & u^{\prime }\leq 0,\;w^{\prime }>0\\ 3, & u^{\prime }<0,\;w^{\prime }\leq 0\\ 4, & u^{\prime }\geq 0,\;w^{\prime }<0 \end{array}\right..$$

These 4 numbers can be thought as symbols/letters of the alphabet $A=\left\{1,2,3,4\right\}$ with 4 letters. The joint velocity fluctuation time series is transformed to a sequence of time-symbol pairs $\left\{\left(t_{i},s_{i}\right)\;|\;s_{i}\in A,\;s_{i}\neq s_{i+1}\;\mathrm{and}\;i=0,1,2,\ldots \right\}$, denoting the occurrence of the distinct quadrant events at time $t_{i}$. This is a *marked point process*, an extension of the simple point process described in Section 3. In our quadrant-event model, outward interaction ($u^{\prime }>0,\;$$w^{\prime }\geq 0$), ejection ($u^{\prime }\leq 0,\;$$w^{\prime }>0$), inward interaction ($u^{\prime }<0,\;$$w^{\prime }\leq 0$), and sweep ($u^{\prime }\geq 0,\;$$w^{\prime }<0$) are the discrete events. The residence times $T_{i}$’s express the average duration that the system spends in a given quadrant.

**Figure 6:**
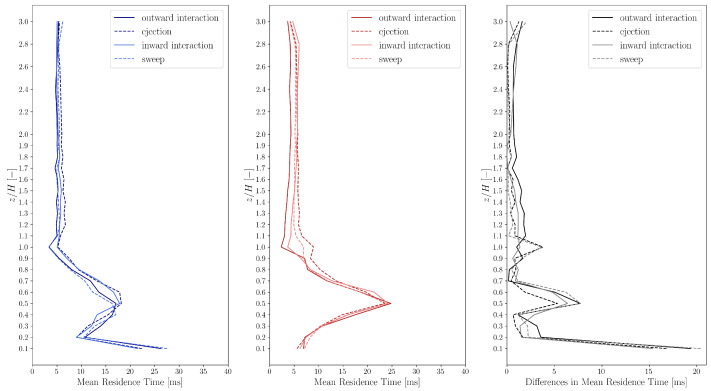
Mean residence times of the “quadrantified” experimental (**left**, blue) and simulation (**middle**, red) data. The differences (**right**, black) between the experimental and simulation data are also shown. Outward interaction: $u^{\prime }>0,w^{\prime }\geq 0$; ejection: $u^{\prime }\leq 0,w^{\prime }>0$; inward interaction: $u^{\prime }<0,w^{\prime }\leq 0$; sweep: $u^{\prime }\geq 0,w^{\prime }<0$.

The comparison of the mean residence times for all 4 events are shown in Figure 6. The mean residence time are 2 ms $<T_{mean}<30$ ms for both cases. In case of the simulation, the mean residence time has a higher peak at z/H=0.5 than for the experimental data, which can be explained by the fact that in the simulation the vortex core is substantially more enclosed than what was observed in the experiment. Corresponding evidence can be found in the concentration plots shown in Papp et al. [53].

**Figure 7:**
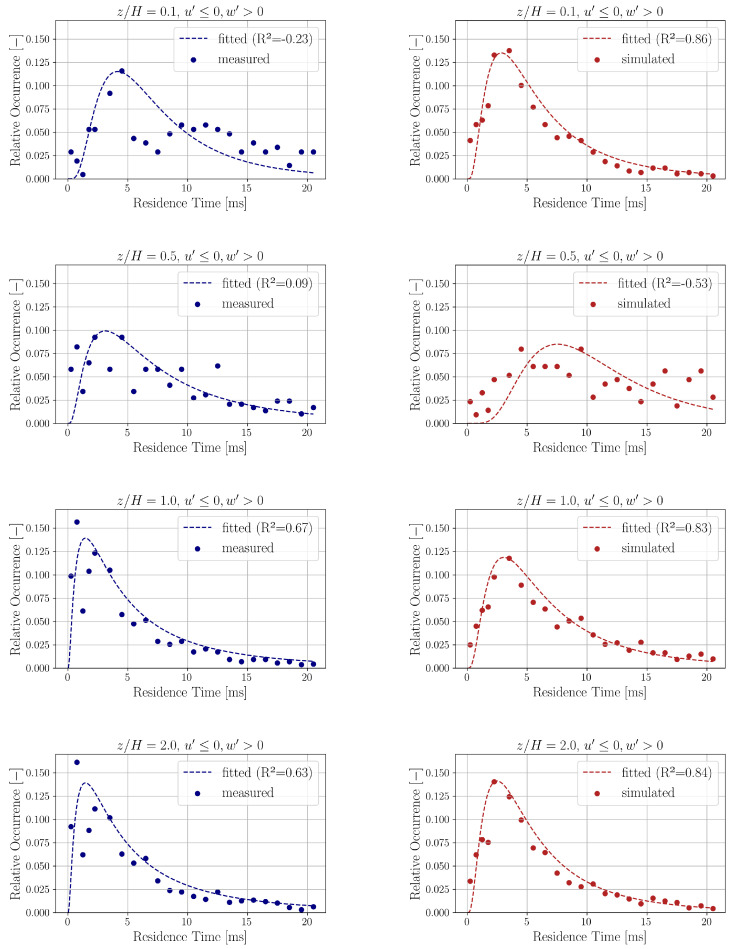
Relative occurrence of ejection ($u^{\prime }\leq 0,\;w^{\prime }>0$) in the experimental (**left**, blue) and the simulation (**right**, red) data measured at different z/H heights.

Figure 7 shows the distribution (relative occurrence) of ejection ($u^{\prime }\leq 0,\;w^{\prime }>0$) residence times for both the experimental and simulation data at heights z/H=0.1,0.5,1.0 and 2.0. The relative occurrences of residence time intervals up to 20 ms are plotted. Bogard et al. [67] reported that the time intervals between ejection events follow a distribution that is approximately exponential. However, several studies have shown that the durations between mean-level crossings in turbulent signals are better described by a lognormal distribution [68,69,70,71]. Based on this observation, we fit the following lognormal probability density function (PDF) to the distribution of relative occurrences of residence times $T_{\mathrm{res}}$

(8) $$P\left(T_{res}\right)=\frac{1}{z_{2}T_{res}\sqrt{2\pi }}\exp\left(-\frac{1}{2}{\left(\frac{\ln T_{res}-z_{1}}{z_{2}}\right)}^{2}\right).$$

where $P(T_{\mathrm{res}})$ denotes the relative frequency associated with residence times $T_{\mathrm{res}}$ across the four quadrant event types (outward interaction, ejection, inward interaction, sweep), and $z_{1}$ and $z_{2}$ are the parameters of the lognormal distribution. The fitted values of these parameters are presented in Table 3.

The fitted lognormal curves are also shown in Figure 7 with their coefficient of determination [72]

(9) $$R^{2}=1-\frac{\sum_{i}{\left(y_{i}-P_{i}\right)}^{2}}{\sum_{i}{\left(y_{i}-\bar{y}\right)}^{2}}.$$

where $y_{i}$ values are the calculated relative occurrence values of residence time $T_{res}$ and $\bar{y}$ is the mean of the relative occurrence values $y_{i}$’s. The predicted relative occurrence values $P_{i}$ are obtained from the fitted lognormal model described in Equation (8). It is important to note that the coefficient of determination $R^{2}$ can occasionally be negative. This occurs when the mean value $\bar{y}$ provides a better fit to the data than the nonlinear model predictions $P_{i}$, as indicated by Equation (9).

The distributions for the other quadrants are qualitatively similar to the ones shown in Figure 7 for ejection. The relative occurrence results show fairly good qualitative agreement between the experimental and simulation data in all analyzed measurement points.

**Figure 8:**
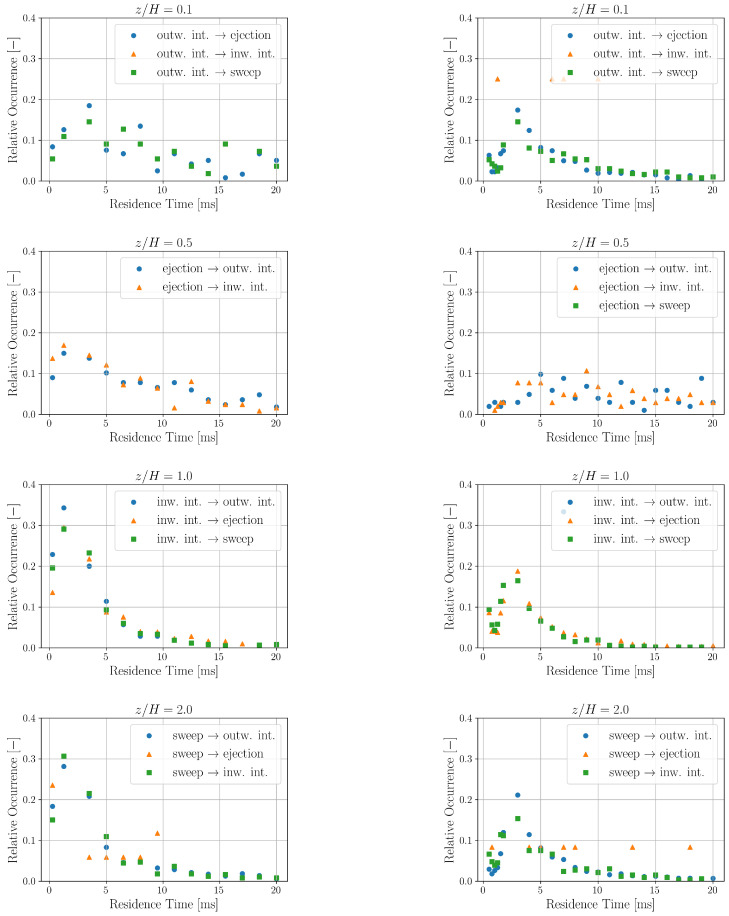
Relative occurrence of one quadrant transitioning into another specific quadrant in the experimental (**left**) and simulation (**right**) data measured at different z/H heights.

The relative occurrences of residence times for a quadrant transitioning to another specific quadrant is shown in Figure 8. The residence time distributions corresponding to one quadrant transitioning to another specific quadrant obtained here are comparable with the ones obtained in [43].

**Figure 9:**
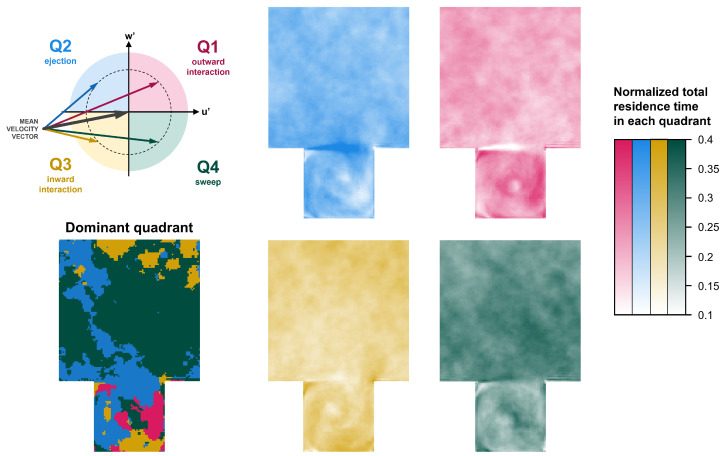
Quadrant statistics based on the CFD results averaged over time and the spanwise direction. The total residence time in each quadrant is normalized by the total residence time in all quadrants combined; therefore, the normalized value must be between 0 and 1. The dominant quadrant is the one with the highest total residence time at each location.

Finally, let us utilize the fine spatial resolution of the CFD simulations to gain further insight into the flow field. Figure 9 shows the quadrant statistics based on the CFD simulations, averaged in time and in the spanwise direction, i.e., over the length of the street canyon. The presence of the shear layer at roof height is clearly visible in the quadrant statistics: at z/H=1.0, the normalized total residence time (probability) corresponding to Q2 (ejection) peaks at a value of above 0.4. Correspondingly, the probabilities of the other quadrants must decrease locally. Interestingly, Q1 (outward interaction) shows a probability below 0.1 in the shear layer. This, combined with the fact that the mean vertical velocity is around zero in the shear layer ($\bar{w}=0$, see Figure 3), means that while downward motion ($w^{\prime }<0$) is equally distributed over Q3 (inward interaction) and Q4 (sweep), upward motion ($w^{\prime }>0$) dominantly happens in Q2 (ejection). In other words, air parcels (or transported pollutants) are likely to escape the canyon when the streamwise velocity component in the shear layer is lower than its time-average.

Furthermore, let us observe the path of a fluid parcel through the clockwise-rotating canyon vortex. Importantly, due to the fact that the mean velocity vector can significantly deviate from the positive *x* direction below roof height, the meaning of the quadrants must be assessed with special attention. Around the 3 o’clock position in the downward-moving fluid ($\bar{u}\approx 0$, $\bar{w}<0$), Q1 is dominant (to the detriment of Q2), meaning that the most probable state of the current velocity vector is of a smaller magnitude than the mean velocity vector, and it is pointing outwards. Further ahead, around the 6 o’clock position, i.e., in the street-level backflow ($\bar{u}<0$, $\bar{w}\approx 0$), Q3 is dominant, and in the upward-moving fluid ($\bar{u}\approx 0$, $\bar{w}>0$) around the 9 o’clock position, the most probable quadrants are Q3 and Q2, respectively. These findings indicate that outward drift is the most likely state in these regions too.

## 5. Word Statistics and Entropy

Following the approach described by Daw et al. [36], symbolic sequences *S*, composed of individual symbols $s_{i}$, are transformed into sequences of words of fixed length *L*. This transformation is accomplished by sliding a window of length *L* across the original sequence.

In formal terms, the sequence *S* is transformed into a multiset of words $W^{L}$ as follows:

(10) $$W^{L}={\left\{w_{i}\right\}}^{\left|S\right|-L+1}=\left\{w_{1},w_{2},\cdots ,w_{\left|S\right|-L+1}\right\}=\left\{s_{1}s_{2}\ldots s_{L},s_{2}s_{3}\ldots s_{L+1},\ldots ,s_{\left|S\right|-L+1}s_{\left|S\right|-L+2}\ldots s_{\left|S\right|}\right\}.$$

The word list $W^{L}$ has $n_{L}=\left|W^{L}\right|=\left|S\right|-L+1$ elements and is a multiset, i.e., it can contain identical words multiple times. We denote with ${\hat{W}}^{L}$ the set of unique words in $W^{L}$ (obtained by removing duplicate words $W^{L}$), with cardinality denoted by ${\hat{n}}_{L}=\left|{\hat{W}}^{L}\right|$.

**Figure 10:**
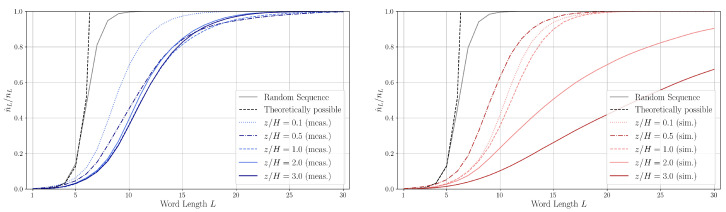
Dimensionless word number ${\hat{n}}_{L}/n_{L}$ calculated from the number of unique words ${\hat{n}}_{L}$ found in the word list $W_{L}$ of measured (**left**, blue), simulated (**right**, red), and random generated symbolic sequences for different word length *L* at different measurement heights. The number of words in word list $W_{L}$ is $n_{L}$.

In Figure 10, the normalized quantities (dimensionless word number) ${\hat{n}}_{L}/n_{L}$ are shown for different heights. For word lengths approximately L=12, the number of unique words ${\hat{n}}_{L}$ in the measured series is approximately the half of the length $n_{L}$ of the measured word lists. This is different for the simulation data, especially for heights z/H=2.0 and 3.0 in the simulation data. We attribute this to the driving force being applied at height z/H=2.0 and the slip symmetry boundary condition at z/H=3.0.

For comparison, the dimensionless word number for both infinite (theoretically possible) and finite random sequences is also shown in Figure 10. One can see that the dimensionless word number curve of the measurement at z/H=0.1, i.e., close to the ground in the canyon, is in-between the curve of the random sequence and the sequences corresponding to the other measurement heights. The random sequence represents a fully disordered, uncorrelated flow; hence, the data above z/H=0.1 may show the presence of coherent structures.

A comparison of the symbolic sequences up to word length L=3 reveals that the number of unique words increases at a nearly identical rate for both measured and randomly generated sequences. However, beyond L=3, the number of unique words in the random sequences grows significantly faster. Due to the finite length of the sequences (an infinite sequence is also shown for comparison), the growth of unique words is naturally bounded by the total number of possible words $n_{L}$. This saturation occurs at *L* = 9–10 for the random sequence, between *L* = 16–30 for the measured sequence and around *L* = 20–50 for the simulated sequences.

**Figure 11:**
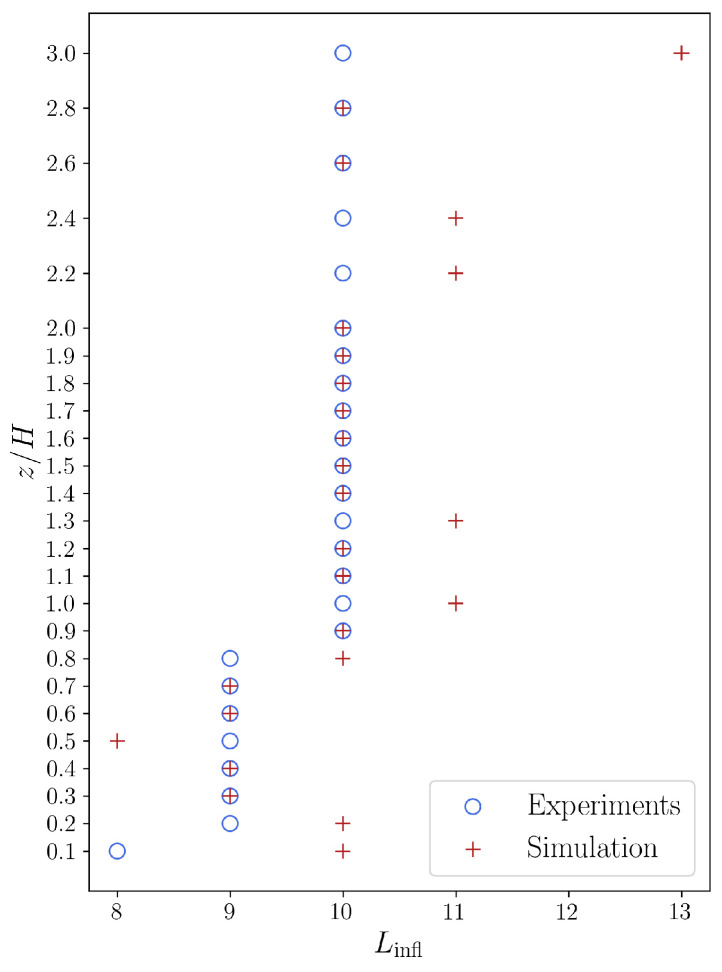
Word length positions of inflection points (blue—experiment, red—simulation) of the dimensional word number curves (see Figure 10).

Figure 11 shows at which word length value *L* is the inflection point of the experimental and dimensionless word number curves. We can state that the inflection points for the experimental and simulation sequences show a good agreement setting aside a few “jumps” in the inflection points of the simulation sequences. We believe the shift of inflection point outside the canyon means that the symbolic sequence—exhibiting a more ordered flow—is diverging from the reference random sequence, i.e., the number of unique symbolic words increases more slowly with the word length.

**Figure 12:**
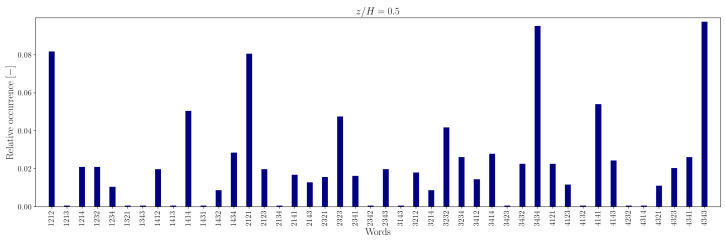
Relative occurrence of unique words of length L=4 in the experimental data at measurement height z/H=0.5.

**Figure 13:**
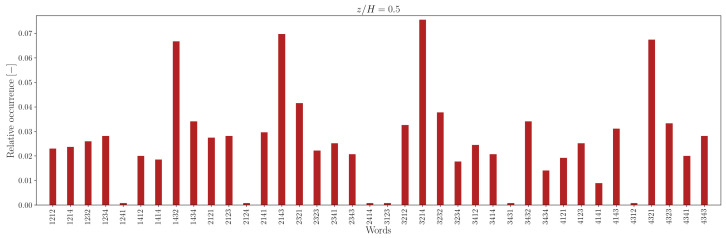
Relative occurrence of unique words of length L=4 in the simulation data at height z/H=0.5.

**Figure 14:**
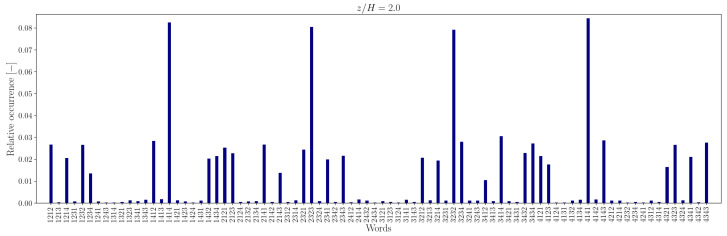
Relative occurrence of unique words of length L=4 in the experimental data at measurement height z/H=2.0.

**Figure 15:**
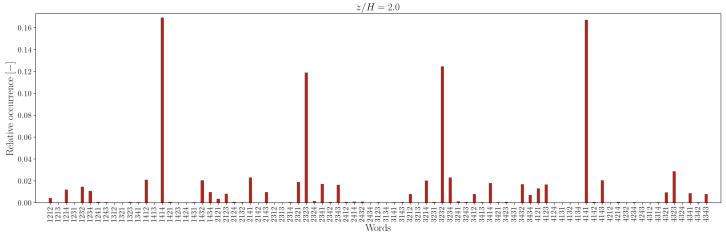
Relative occurrence of unique words of length L=4 in the simulation data at height z/H=2.0.

The lower number of unique words in the measured sequence, relative to the random counterpart, is attributable to the recurrence of specific words, indicating that some word patterns appear with higher frequency. For instance, the relative frequency distribution of words of length L=4 is illustrated in Figure 12, Figure 13, Figure 14 and Figure 15.

In the canyon at z/H=0.5, the four most common words are 1212/2121 and 3434/4343 in the experimental sequence and 1432/3214 and 4321/2143 in the simulation sequence. Outside the canyon, for both experimental and simulation data, the four most common words are 1414/4141 and 2323/3232.

At longer word lengths, the most frequently observed words are in which symbol pairs 12/21 and 34/43 (within the canyon) and 14/41 and 23/32 (outside the canyon) follow one another. These alternating patterns indicate that pairs of outward interaction–ejection events (1↔2) and inward interaction–sweep events (3↔4) commonly follow one another inside the canyon, possibly due to the influence of the no-slip boundary condition at the canyon bottom.

In contrast, outside the canyon, the repetition of symbol pairs 1,4 and 2,3 suggests a tendency for outward interaction–sweep and ejection–inward interaction pairs to alternate. This behavior implies that the reduced quadrantified signal often becomes trapped in cycles involving rapid switching between two dominant turbulent event states.

Note that moving away from the bottom of the canyon, the most common unique words represent almost 40−50% of the word list. The word frequency distributions show a somewhat central symmetry that suggests a self-similar (multi-fractal) nature of the data [73].

We characterize the degree of regularity and information content within the measured reduced symbolic sequences using the normalized entropy $H_{\mathrm{norm}}^{L}$, a modified form of Shannon entropy as introduced by Finney et al. [39]. For a given word length *L*, it is defined as:

(11) $$H_{\mathrm{norm}}^{L}=-\frac{1}{\ln\left({\hat{n}}_{L}\right)}\sum_{1}^{n_{L}}P\left(w_{i}\right)\ln P\left(w_{i}\right).$$

where $P(w_{i})$ represents the relative frequency of the word $w_{i}$ in the word list $W^{L}$. Notably, this formulation parallels the concept used in Kolmogorov–Sinai entropy for evaluating the information rate in dynamical systems. A review on entropy in various fields of science is given in [74].

**Figure 16:**
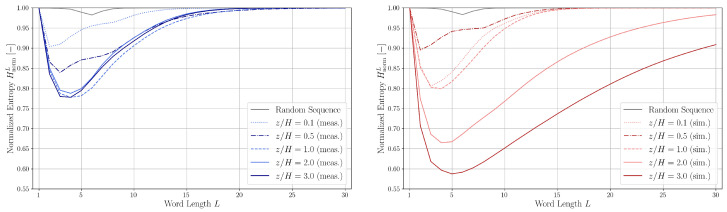
Normalized entropy $H_{\mathrm{norm}}^{L}$ as a function of word length *L* for experimental (**left**, blue) data and simulation (**right**, red) data at different measurement heights and for a random sequence.

The normalized entropy values for various word lengths and heights are plotted in Figure 16.

The entropy curves exhibit trends consistent with those reported by Finney et al. [39]: a significant decrease in entropy occurs for the shortest word lengths, reaching a minimum around *L* = 2–5, followed by a gradual approach toward $H_{\mathrm{norm}}^{L}=1$ at larger *L*.

Higher entropy values indicate greater disorder and randomness, whereas lower entropy suggests more correlated, deterministic behavior, the dip of entropy suggest the presence of structured patterns or intermittency. For the random sequence, $H_{\mathrm{norm}}^{L}$ remains close to 1 across all word lengths, reflecting the absence of meaningful structure. The small dip near L=6 is attributed to the finite length of the random dataset.

The smallest entropy value is obtained at z/H=1.0,3.0 for the measurement data and at z/H=2.0,3.0 for the simulation data. The entropies of the experimental and simulation sequences at z/H=1.0 show good agreement; at z/H=0.5, the flow is more disordered in the simulation data, and at z/H=2.0 and 3.0, one can again see the effect of driving force and slip symmetry boundary condition the makes the flow more ordered than under real-world conditions.

## 6. Surrogate Series

In Section 5, we compare the normalized entropy $H_{\mathrm{norm}}^{L}$ derived from symbolic analysis of measured and simulated velocity fluctuation data, as well as from a random sequence. To assess the variation of entropy as a function of word length across different types of symbolic sequences, we generated the following artificial sequences, all having the same length as the measured dataset:

- **Noisy periodic sequence:** A periodic symbolic pattern constructed by repeating the block {1,3,1,3,2,3,1,4}, with a subset of symbols randomly replaced at a controlled rate. Replacements are constrained to differ from both the preceding and following symbols.
- **Markov chain-generated sequences:** Symbolic sequences generated using Markov models of orders up to five, constructed from transition probabilities estimated from both measured and simulated data.

To construct these Markov-based surrogate sequences, we define transition probabilities between quadrant states. Transitions between the four quadrants are categorized as follows: *stable* (remaining in the same quadrant), *cross* (transitions such as 1↔3, 2↔4), and *marginal* (transitions such as 1↔2, 3↔4). Kalmár-Nagy and Varga [43] observed that stable transitions have the highest probability. This finding is in agreement with earlier observations by Keshavarzi et al. [75,76], who identified similar patterns of transition preference near different types of boundaries, including plain beds, ripple beds, and obstacles such as bridge piers.

When the time stamps of events are discarded from the marked point process, the result is a sequence of symbolic words of length *L*, each composed of four-symbol combinations $s_{i}s_{i+1}\ldots s_{i+L-1}$. A key constraint on these sequences is that no two adjacent symbols are identical, i.e., $s_{i}\neq s_{i+1}$.

If the symbolic sequence arises from a Markovian process, transitions between symbols can be characterized by transition probabilities $p_{i\to j}$ (denoted simply as $p_{ij}$). Under this assumption, the symbolic quadrant sequence *S* forms a first-order Markov chain with the transition probability matrix:

(12) $$M^{1}=\left(\begin{array}{cccc} p_{11} & p_{12} & p_{13} & p_{14}\\ p_{21} & p_{22} & p_{23} & p_{24}\\ p_{31} & p_{32} & p_{33} & p_{34}\\ p_{41} & p_{42} & p_{43} & p_{44} \end{array}\right).$$

where $p_{ij}$ denotes the probability of transitioning from quadrant *i* to quadrant *j*. For our quadrant-based symbolic process, self-transitions are not permitted, so the diagonal elements $p_{11},p_{22},p_{33},p_{44}$ are all zero. These stable movements instead correspond to the residence times spent within each state.

To generalize the size of the transition matrix for a Markov model of order *m*, we consider an alphabet *A* of λ symbols. The number of valid symbolic words of length *L*, where adjacent symbols differ, is given by:

(13) $$|W_{\lambda }^{\mathrm{pos}}{|=\lambda \left(\lambda -1\right)}^{L-1}.$$

In our case with λ=4, this evaluates to $|W_{4}^{\mathrm{pos}}|=4\cdot 3^{L-1}$.

The elements of the transition probability matrix $M^{m}$ for a Markov chain of order m=L−1 are defined as follows:

(14) $$M_{s_{j-L+1}\ldots s_{j-2}s_{j-1}\to s_{j}}^{m}=p\left(s_{j}=i|s_{j-L+1}\ldots s_{j-2}s_{j-1}\right),\;j=L-1,\ldots ,\left|W_{\lambda }^{\mathrm{pos}}\right|,\;i=1,\ldots ,\lambda .$$

This expression specifies the conditional probability that a symbol $s_{j}=i$ follows the prefix sequence $s_{j-L+1}\ldots s_{j-1}$ within a valid word $w_{j}\in W^{\mathrm{pos}}=\left\{w_{i}^{\mathrm{pos}}\right\}$.

**Figure 17:**
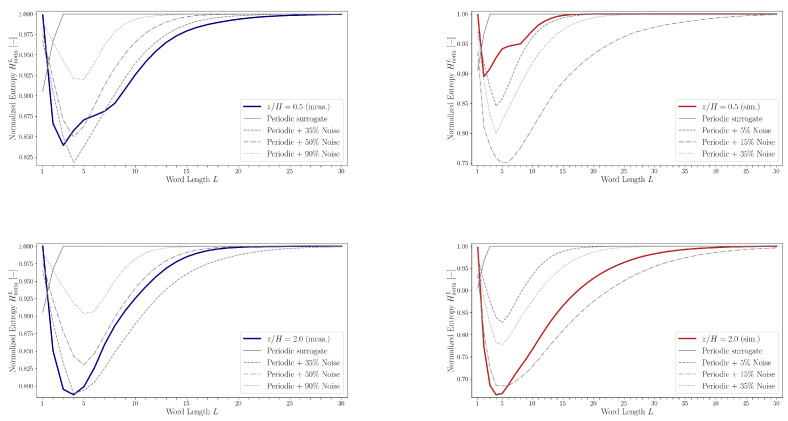
Comparisons of normalized entropy $H_{norm}^{L}$ belonging to the experimental (blue)/simulation (red) data and the artificial noisy periodic sequences as the function of word length *L*.

Figure 17 presents a comparison of normalized entropy values $H_{\mathrm{norm}}^{L}$ as a function of word length *L* for both the measured and artificially generated noisy periodic sequences. For the purely periodic sequence, $H_{\mathrm{norm}}^{L}$ remains close to 1 across nearly all word lengths. This behavior is attributed to the nearly uniform distribution of words in the partitioned word list $W^{L}$, which mimics the properties of a random sequence where all unique words have approximately equal probability of occurrence.

Entropy curves for several noisy periodic sequences, derived by corrupting the base periodic pattern with noise levels of 35%, 50%, and 90% (relative to the length of the measured sequence) and 5%, 15%, and 35% (relative to the length of the simulated sequence), are also shown in Figure 17. These entropy profiles exhibit characteristics similar to those observed in measured and simulated data: they are unimodal and gradually converge toward $H_{\mathrm{norm}}^{L}=1$. Additionally, the location of the entropy minimum shifts to higher word lengths with increasing noise. However, the entropy minima for the experimental and simulated datasets tend to occur at shorter word lengths compared to those of the noisy periodic sequences.

**Figure 18:**
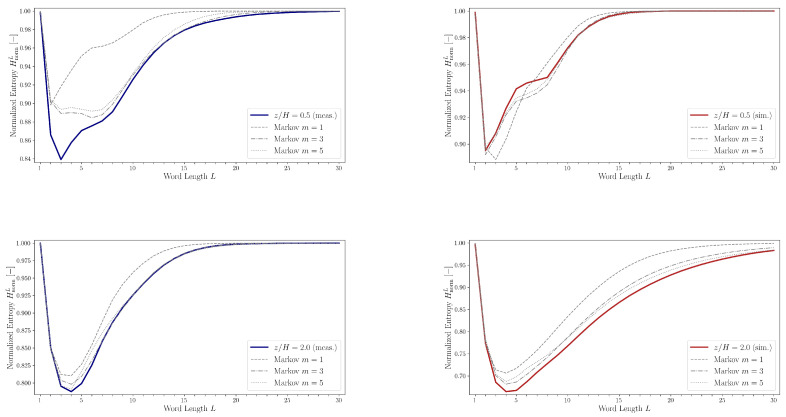
Comparisons of normalized entropy $H_{norm}^{L}$ belonging to the experimental (blue)/simulation (red) data and the artificial sequences generated using Markov chains (transition probabilities obtained from the experimental/simulation data).

The transition probability matrices $M^{m}$ were computed up to order m=5 (see Equation (14)) using experimental and simulation data at heights z/H=0.5 and z/H=2.0. These matrices were then used to generate surrogate symbolic sequences. The corresponding normalized entropy values $H_{\mathrm{norm}}^{L}$ for both the original and the surrogate sequences are compared in Figure 18.

In general, surrogate sequences generated using fifth-order Markov models provide the closest match to the entropy curves of the measured and simulated data. An exception arises at z/H=0.5 in the experimental dataset, where the characteristic dip in the entropy curve is not replicated even by the fifth-order Markov chain. This discrepancy suggests that the symbolic quadrant sequence possesses a level of temporal correlation or structural complexity that exceeds what can be captured by a fifth-order Markov process.

Such behavior points to the influence of temporally coherent structures within the flow field, and supports the interpretation that the quadrant series retains memory effects over longer word lengths, indicative of organized turbulence phenomena.

## 7. 2D Distributions of Velocity Fluctuations

**Figure 19:**
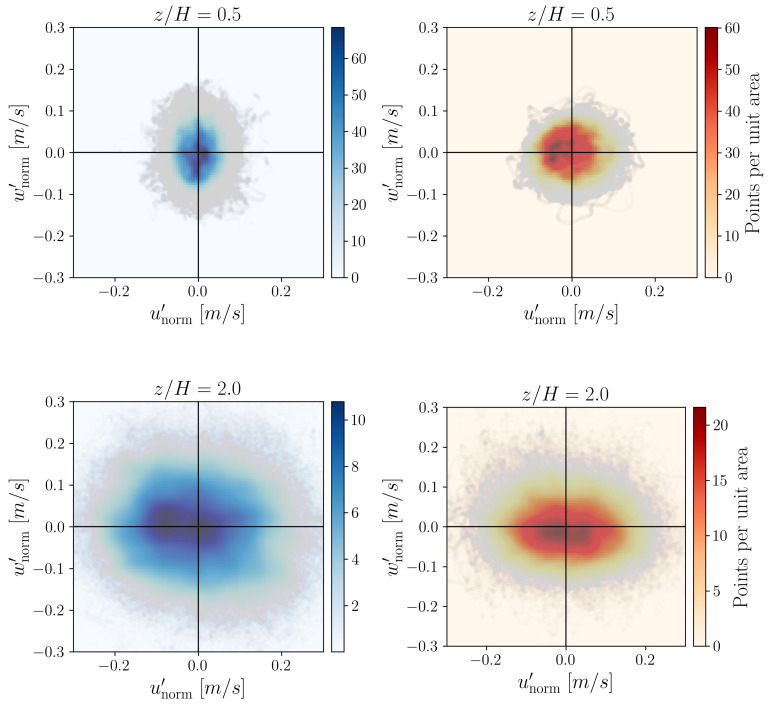
Velocity fluctuation pairs $\left(u_{\mathrm{norm}}^{\prime },w_{\mathrm{norm}}^{\prime }\right)$ and their probability density distribution of the fluctuation pairs at heights z/H=0.1 (**first row**) and z/H=2.0 (**second row**). The experimental data on the left are denoted by blue and the simulation data on the right with red.

Figure 19 shows the velocity fluctuation pairs $\left(u_{\mathrm{norm}}^{\prime },\;w_{\mathrm{norm}}^{\prime }\right)$ normalized with the free stream velocity $u_{\infty }=9$ m/s colored by the probability density distribution of the fluctuation pairs at heights z/H=0.5 and 2.0. The probability distributions were calculated by the Gaussian kernel-density estimation utilizing a 100×100 grid over the point cloud range. The probability distributions of the point clouds are colored by point density.

Comparing the probability distribution in Figure 19 to the relative occurrence of words in Figure 12, Figure 13, Figure 14 and Figure 15, one can see that for z/H, the “horizontal” transitions 1↔2 and 3↔4 are really more frequent at height z/H=5 in the experimental data. However, for larger heights like z/H=2.0, the transitions 1↔4 and 2↔3 happen more. This means that while the fluctuations may display isotropy at some point, at the investigated heights, there is a preferential orientation of the velocity fluctuations. A comparable pattern of anisotropy was reported in open-channel flow over a rough bed by [20].

**Figure 20:**
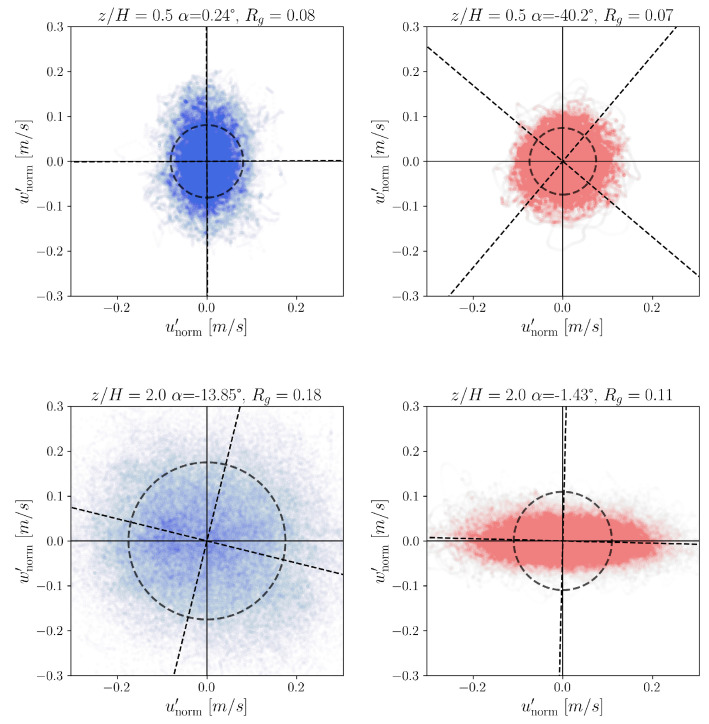
Velocity fluctuation pairs $\left(u_{\mathrm{norm}}^{\prime },w_{\mathrm{norm}}^{\prime }\right)$ and their probability density distribution of the fluctuation pairs at heights z/H=0.1 (**first row**) and z/H=2.0 (**second row**). The experimental data on the left are denoted by blue and the simulation data on the right with red. The angle between the vertical axis and the principal axis of the point cloud is denoted by α. The values of the radii of gyration $R_{g}$ are also given.

Note that at z/H=2.0 the distribution of the simulation data shows a remarkably flat ellipse compared to the experimental result. This also highlights that there is a noticeable deficit in the vertical fluctuations above roof height, a finding consistent with the results shown in Figure 3, and is a shortcoming of the applied model (also shown in Figure 20).

For all velocity fluctuation pairs $\left(u_{\mathrm{norm}}^{\prime },w_{\mathrm{norm}}^{\prime }\right)$ the moment of inertia matrices,

(15) $$I=\left(\begin{array}{cc} \sum_{1}^{N}w_{\mathrm{norm}}^{\prime^{2}} & \sum_{1}^{N}u_{\mathrm{norm}}^{\prime }w_{\mathrm{norm}}^{\prime }\\ \sum_{1}^{N}u_{\mathrm{norm}}^{\prime }w_{\mathrm{norm}}^{\prime } & \sum_{1}^{N}u_{\mathrm{norm}}^{\prime^{2}} \end{array}\right)$$

determines the principal axis (dashed straight lines in Figure 20) of the point clouds. The angles between the $u^{\prime }$ axis and the principal axis relative to $u^{\prime }$ are $\alpha_{0.5}=0.24^{{}^{\circ}}$ and $\alpha_{2.0}=-13.85^{{}^{\circ}}$ for the experimental data and $\alpha_{0.5}=-40.20^{{}^{\circ}}$ and $\alpha_{2.0}=-1.43^{{}^{\circ}}$ for the simulation data. Obviously, the calculated angles for both cases are different, showing the difference between a vortex core at z/H=0.5 in the CFD model and the measurement, as well as the effect of driving force at z/H=2.0.

From the inertia matrices, the radii of gyration $R_{g}$ were determined, too, for each measurement and simulation height (represented by the dashed circles in Figure 20). The radii of gyration values show better agreement for the experimental and simulation data.

Since the proper partitioning for the use of symbolic dynamics is still an open question [77], we also investigated the influence of coordinate rotation on the entropy. We considered the coordinate system $\left(u^{\prime *},w^{\prime *}\right)$ generated from the original $\left(u_{\mathrm{norm}}^{\prime },w_{\mathrm{norm}}^{\prime }\right)$ system by a counterclockwise rotation by angle θ as

(16) u′*w′*=Rθu′w′,

(17) Rθ=cosθ−sinθsinθcosθ.

For a given rotation angle θ, the rotation (16) is applied to each pair of normalized velocity fluctuations $\left(u_{\mathrm{norm}}^{\prime },w_{\mathrm{norm}}^{\prime }\right)$. The resulting rotated fluctuation components $\left(u^{\prime *},w^{\prime *}\right)$ are then classified into symbolic sequences through quadrant analysis, yielding the series $\left\{Q\left(u^{\prime *},w^{\prime *}\right)\right\}$.

**Figure 21:**
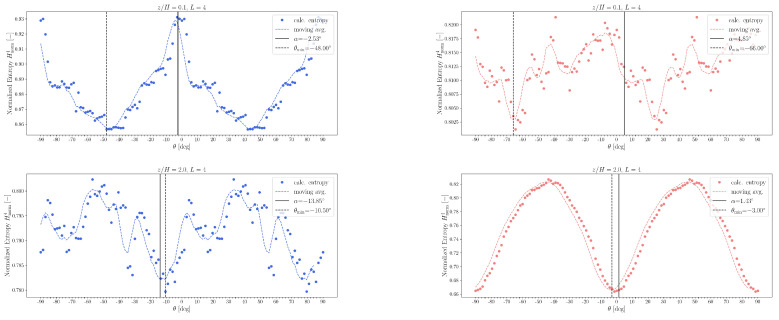
$H_{norm}^{4}$ normalized entropy values for word length L=4 at heights z/H=0.1 and 2.0. On the left, blue figures correspond to the experimental data, while on the right, red figures correspond to the simulation data.

Using this procedure, the normalized entropy $H_{\mathrm{norm}}^{4}$ (see Equation (11)) was computed as a function of the rotation angle θ. Figure 21 displays the resulting entropy profiles for measurement heights z/H=0.1 and z/H=2.0, with angular resolution $\Delta \theta =1.5^{{}^{\circ}}$.

As expected, the function $H_{\mathrm{norm}}^{4}\left(\theta \right)$ exhibits π/2-periodicity because a $\pm 90^{{}^{\circ}}$ rotation simply permutes the quadrant labels. Kalmár-Nagy and Varga [43] observed that the locations of entropy extrema with respect to θ are invariant under changes in word length. They further reported that the rotation angle $\theta_{\min}$, corresponding to the minimum of the smoothed entropy curve (indicated by vertical dashed lines), tends to align closely with the principal axis directions (marked by vertical solid lines). However, in our case, this holds only outside the canyon region.

The hypothesis of Kalmár-Nagy and Varga was that the observed entropy minimum results from the most frequent transitions. In the original coordinate system, these transitions primarily cross the $u_{\mathrm{norm}}^{\prime }$ axis. However, transitions more frequently align with the principal axis, so when the system is rotated accordingly, the number of transitions increases, leading to lower entropy values. This interpretation, however, does not hold at height z/H=0.1, as evident from Figure 21.

## 8. Conclusions

We analyzed and compared time series data from wind tunnel measurement and CFD simulation of flows over street canyons through the use of the quadrant method, calculating entropies, Markov matrices and generating surrogate series.

We studied the idea that coherent structures in the atmospheric boundary layer manifest as “almost periodic temporal patterns”, characterized by the imperfect repetition of specific turbulent events or states. The time series of fluctuating velocities were converted into symbolic sequences using the quadrant method and treated as marked point processes. Analysis of the residence times—defined as the duration spent in each quadrant state—revealed distributions consistent with a lognormal form. To evaluate the information content of the symbolic sequences, normalized entropy was calculated across a range of word lengths. The resulting entropy profiles and the number of unique symbolic words were then compared to those derived from random sequences. Interestingly, artificially generated noisy periodic sequences produced entropy trends that qualitatively matched those of the experimental data.

Furthermore, surrogate sequences were created using Markov processes (orders 1 through 5). These surrogates reproduced entropy distributions close to those of the measured and simulated series, supporting the suitability of higher-order Markov models for characterizing the observed symbolic dynamics. Analysis of symbolic word histograms indicated that much of the information content in both measured and simulated sequences arises from the elevated frequency of words composed of alternating pairs of symbols.

Our investigation showed that simulation results are in qualitative agreement with measurements in several aspects, including the general trends observed in the residence time distributions of quadrants, the inflection points of dimensionless word number curves, as well as word frequencies. The vortex structures within the canyon, as the 2D distributions of the velocity fluctuations show, are well captured, demonstrating the capability of the model to reproduce the dominant flow patterns. It should be noted that some discrepancies exist, such as the differences in the mean residence time of the states above roof level (z/H>1.0) and in the entropy minima. Another discrepancy is the underestimation of the vertical velocity fluctuation ($w^{\prime }$) magnitude, but it is a known imperfection of the simulation model used in this work. Note that an important advantage of the CFD model used in the present work is that it provides a valuable tool for analyzing the entire flow field across the computational domain, offering insight beyond the discrete measurement points.

## Figures and Tables

**Table 1:** Summary of the numerical simulations carried out at three different spatial resolutions.

Parameters of Numerical Meshes	Coarse	Medium	Fine
Mesh resolution (Δx=Δx=Δz)	H/22	H/32	H/48
Total cell count (N)	111k	343k	1312k
Time step size (Δt)	0.45 ms	0.3 ms	0.2 ms
Total number of data points $\left(T_{s}/\Delta t\right)$	67k	100k	150k

**Table 2:** Statistical properties of $u\left(t\right)$ and $w\left(t\right)$ from experimental (Exp.) and simulation (Sim.) data.

** u(t) **												
	**Min [m/s]**		**Mean [m/s]**		**Max [m/s]**		** σ [m/s] **		**Skewness** [−]		**Kurtosis** [−]	
**Location**	**Exp.**	**Sim.**	**Exp.**	**Sim.**	**Exp.**	**Sim.**	**Exp.**	**Sim.**	**Exp.**	**Sim.**	**Exp.**	**Sim.**
z/H=0.1	−3.588	−3.293	−1.574	−2.119	0.011	−1.124	0.531	0.261	−0.387	−0.109	0.320	0.332
z/H=0.5	−1.862	−1.092	0.013	0.021	1.584	1.275	0.418	0.348	0.037	0.151	0.157	−0.102
z/H=1.0	−0.131	1.088	2.494	2.138	6.744	5.850	0.992	0.425	0.716	1.350	0.183	3.930
z/H=2.0	1.373	2.360	5.015	5.078	9.815	8.260	1.296	0.933	0.131	0.062	−0.577	−0.334
z/H=3.0	2.877	3.587	7.008	5.676	10.66	8.086	1.222	0.666	−0.378	0.054	−0.205	−0.287
** w(t) **												
	**Min [m/s]**		**Mean [m/s]**		**Max [m/s]**		** σ [m/s] **		**Skewness** [−]		**Kurtosis** [−]	
**Location**	**Exp.**	**Sim.**	**Exp.**	**Sim.**	**Exp.**	**Sim.**	**Exp.**	**Sim.**	**Exp.**	**Sim.**	**Exp.**	**Sim.**
z/H=0.1	−2.176	−1.589	−0.027	0.014	1.707	1.370	0.474	0.318	−0.138	0.254	0.878	0.840
z/H=0.5	−1.972	−1.210	−0.072	−0.041	2.029	0.943	0.664	0.311	0.067	−0.073	−0.442	0.052
z/H=1.0	−3.329	−1.364	−0.097	0.014	3.538	1.125	0.721	0.322	−0.036	−0.364	0.762	−0.067
z/H=2.0	−3.541	−0.933	−0.043	−0.014	5.237	1.438	1.074	0.267	0.055	0.408	−0.175	0.466
z/H=3.0	−3.315	−0.185	0.124	0.001	3.956	0.189	0.960	0.030	−0.044	0.490	0.251	2.212

**Table 3:** Lognormal fit parameters for residence time distributions belonging to ejection ($u^{\prime }\leq 0,\;w^{\prime }>0$) at different heights.

	$u^{\prime }\leq 0,\;w^{\prime }>0$			
	$z_{1}$		$z_{2}$	
Location	Exp.	Sim.	Exp.	Sim.
z/H=0.1	1.869	1.640	0.668	0.769
z/H=0.5	1.908	2.311	0.878	0.539
z/H=1.0	1.560	1.757	1.085	0.798
z/H=2.0	1.560	1.575	1.085	0.814

## Data Availability

The data presented in this study are available on request from the corresponding author.
